# Using biomarkers and independent predictors of therapy response to optimize treatment of uncontrolled severe asthma in the biologic era

**DOI:** 10.3389/falgy.2025.1719820

**Published:** 2026-01-09

**Authors:** Hideki Yasui

**Affiliations:** 1 Center for Clinical Research, Hamamatsu University Hospital, Hamamatsu, Japan; 2 Respiratory Medicine, Hamamatsu University School of Medicine, Hamamatsu, Japan

**Keywords:** biologics, biomarkers, independent predictors, review, uncontrolled severe asthma

## Abstract

Severe asthma is a chronic respiratory disease characterized by a lack of control with maximal standard therapy or exacerbation upon therapy reduction. Recent advances in the diagnosis and management of severe asthma have improved patient outcomes. An improved mechanistic understanding of asthma has revealed that many cases are driven by type 2 inflammation, which can be targeted with biologic agents including omalizumab (anti-IgE), mepolizumab and reslizumab (anti-IL-5), benralizumab (anti-IL-5R*α*), dupilumab (anti-IL-4Rα), and tezepelumab (anti-thymic stromal lymphopoietin). Biomarkers, including elevated fractional exhaled nitric oxide, blood eosinophil counts, and serum IgE levels, have been validated for the diagnosis of severe asthma and can be used to help guide disease management. These biologic agents and biomarkers have changed the clinical management of severe asthma, making it possible to pursue the goal of clinical remission. However, despite these advances, a proportion of patients continue to experience uncontrolled severe asthma, which has significant implications for disease management and quality of life. In this review, I briefly examine the current state of biologics and biomarkers in the treatment of uncontrolled severe asthma, and draw on my clinical experience to highlight limitations to optimal management, including persistent treatment heterogeneity. After discussing emerging biomarkers and predictors of disease status and treatment response, I provide my perspective on future approaches and research directions that may enhance clinical treatment and improve patient outcomes.

## 1 Introduction

### 1.1 Immunological mechanisms and targeted biologics

Severe asthma is a chronic respiratory condition caused by inflammation of the airways that cannot be controlled by treatment with the maximal standard therapy, or that is exacerbated upon decreasing high-dose therapy (1). Globally, approximately 300 million individuals have asthma (2). Recent studies suggest the prevalence of severe asthma among all asthma cases may be lower than the previously estimated 5%–10%, at approximately 3%–4% in some European and Japanese cohorts (3–5). However, this figure appears to vary widely by region (6), highlighting one of the areas of heterogeneity associated with severe asthma.

Recent research has revealed the immune pathways involved in many cases of severe asthma. Type 2 immune responses—characterized by high expression of interleukin (IL)-4, IL-5, and IL-13—are associated with asthma featuring elevated blood or sputum eosinophil counts, serum immunoglobulin E (IgE) levels, and fractional exhaled nitric oxide (FeNO) (7). Severe asthma cases without a type 2 phenotype, termed “type 2-low” asthma, are more likely to be neutrophilic or paucigranylocytic (8). There is no consensus definition of type 2-low asthma, but type 2-high asthma can be diagnosed with clinical biomarkers that quantitatively assess type 2 pathway activation, in particular FeNO levels and blood eosinophil counts (9).

The increasingly mechanistic understanding of severe asthma has made it possible to target relevant pathways, rather than simply controlling symptoms (9). Biologics are selected for use in severe asthma based on type 2-high characteristics, usually evaluated by measuring blood eosinophil counts, FeNO, sputum eosinophils, and/or determining that the asthma is driven by allergens (3). Indeed, a recent review summarized the process to identify the appropriate biologic with three questions: whether 1) the condition is truly severe asthma, 2) the asthma is eosinophilic, and 3) it is primarily allergen-driven (10). Further recent reviews have documented how treatment with biologics can help prevent exacerbations, hospitalization, and the need for systemic corticosteroids in patients with severe asthma (8, 9). Articles published recently suggest that biologics can decrease airway remodeling (11), reduce exacerbations and enhance asthma control (12), and improve forced expiratory volume (13).

### 1.2 Shifting goals for severe asthma treatment

An initial asthma diagnosis is made based on an assessment of whether symptoms, patient history, and/or examination findings are consistent with asthma or typical of the condition (3). Further evaluation considers whether the patient is already receiving inhaled corticosteroids, whether their symptoms are uncontrolled, and the results of spirometry tests, if available. Severe asthma occurs in a subset of patients who require high-intensity therapy and have poor symptom control. The Global Initiative for Asthma (GINA) guidelines provide an outline for assessing factors contributing to severe status (3). Furthermore, the GINA guidelines offer approaches to optimize treatment and/or adherence, which should be followed by a period of several months before reviewing asthma status.

A diagnosis of severe asthma may arise if the disease is not controlled by initial treatment optimization. This leads to further investigation of comorbidities and asthma phenotype to determine the best course of further treatment. At this point, if asthma is not controlled and type 2-high inflammation is evident, a type 2-targeted biologic may be indicated. An initial treatment period of 4–6 months is typically followed by assessment to determine the patient response. Classification as uncontrolled severe asthma is based on frequency of exacerbations, poor daily control, and the need for systemic corticosteroids. In clinical practice, the possibility of severe asthma is evaluated with chest x-ray, echocardiography, and chest computed tomography to rule out heart failure and other diseases that may result in airway narrowing. Furthermore, it is necessary to ensure that the use of inhaled therapy is appropriate. Biologics are indicated when differential diseases are ruled out and exacerbations occur, or when symptom control is poor despite the use of appropriate inhalation therapy.

The implementation of biologic treatment for severe asthma has changed the clinical perspective on its management. Therapeutic goals have shifted from short-term symptom relief to the achievement of clinical remission (1, 14). Studies suggest that appropriate management of severe asthma can lead to long-term responses, such that patients do not require oral corticosteroids or experience exacerbations, and exhibit stable lung function (15, 16). Several articles have presented definitions of clinical remission for uncontrolled severe asthma, many of which share common features. Severe asthma remission is typically characterized by symptom control without systemic corticosteroids; improvements in asthma control scores such as those for the Asthma Control Questionnaire, Asthma Control Test, and Asthma Impairment and Risk Questionnaire; and the resolution of inflammation as measured by forced expiratory volume, blood eosinophil count, or FeNO (17). However, definitions of remission, or criteria for complete responders, vary in their specific details. Some definitions focus on clinical remission, whereas others are aimed toward complete remission. Furthermore, not all definitions incorporate the same measures or use the same cut-off values (18). The European Respiratory Society recently published two articles on remission, one in favor of incorporating the concept into severe asthma care (19) and the other highlighting the obstacles to defining and achieving remission in this setting (20). Current definitions vary in their measured outcomes (i.e., functional, inflammatory, or patient-reported), and Eggert et al. (20) concluded that the definitions lack practical applicability. These publications emphasize the work that remains to be done to define remission as an outcome for uncontrolled severe asthma treatment.

Severe asthma is a heterogeneous condition, making it difficult to define clinical remission using a single criterion. However, it may be reasonable to consider one season of no exacerbations, well-controlled asthma-related symptoms, and the ability to live without oral corticosteroids as indicative of remission. The ability to define clinical remission using a single measure is a goal for the future. Crucially, despite advances in treatment, a subset of patients experience uncontrolled severe asthma, with a concomitant diminution of quality of life (21). Indeed, an epidemiological study in Japan revealed that approximately 40% of patients with severe asthma have a status of “uncontrolled,” even in the biologic era (5, 22). Further research is required to more completely understand the mechanisms driving uncontrolled severe asthma, guide optimal therapeutic decision-making, and improve treatment outcomes.

### 1.3 Aim of the article

I aim to provide an expert clinical perspective on the optimization of the treatment of uncontrolled severe asthma with biologics, with a focus on the use of biomarkers and independent predictors, in light of recent findings and persistent treatment heterogeneity. In the article, I will consider emerging biomarkers, independent predictors of disease status and treatment response, and other tools that may help meet existing challenges in the ongoing management of severe asthma with biologics. The need to consider comorbidities, such as chronic sinusitis, will also be highlighted. The review will discuss the importance of understanding patient preferences and patient-reported outcomes, and the use of shared decision-making to guide treatment. Finally, the article will conclude by suggesting future research directions to improve severe asthma treatment.

### 1.4 Literature search methodology

The initial literature search for this narrative review was performed on PubMed from January to February 2,025, for articles published between November 2024 and February 2025. The time range was selected to minimize content overlap with recently published review articles. The search was performed with the term “‘uncontrolled severe asthma’ + biologics + biomarkers,” which returned three results, all of which were selected as references. Thereafter the search phrase “’severe asthma’ + biologics + biomarkers + treatment” yielded 37 results, 28 of which were selected after excluding articles focused on pediatric asthma, non-asthma conditions, pre-clinical/translational studies, or those without data (protocols only). The next search, “’severe asthma’ + biologics + ‘independent predictors’,” returned a single result, which was selected as a reference (a similar search using “uncontrolled severe asthma” yielded no results). Finally, a search for “’severe asthma’ + biologics + predictors” returned eight results, of which three were included that had not previously been selected or excluded. Further articles were selected to support discussion of areas of particular interest, including emerging biomarkers (especially miRNAs), consensus definitions, independent predictors, patient-reported outcomes, and shared decision-making. Supplemental references were accessed from the reference lists of the originally selected articles or through targeted searches to provide complete background information on selected topics.

## 2 Current strategies to treat uncontrolled severe asthma

**Figure 1:**
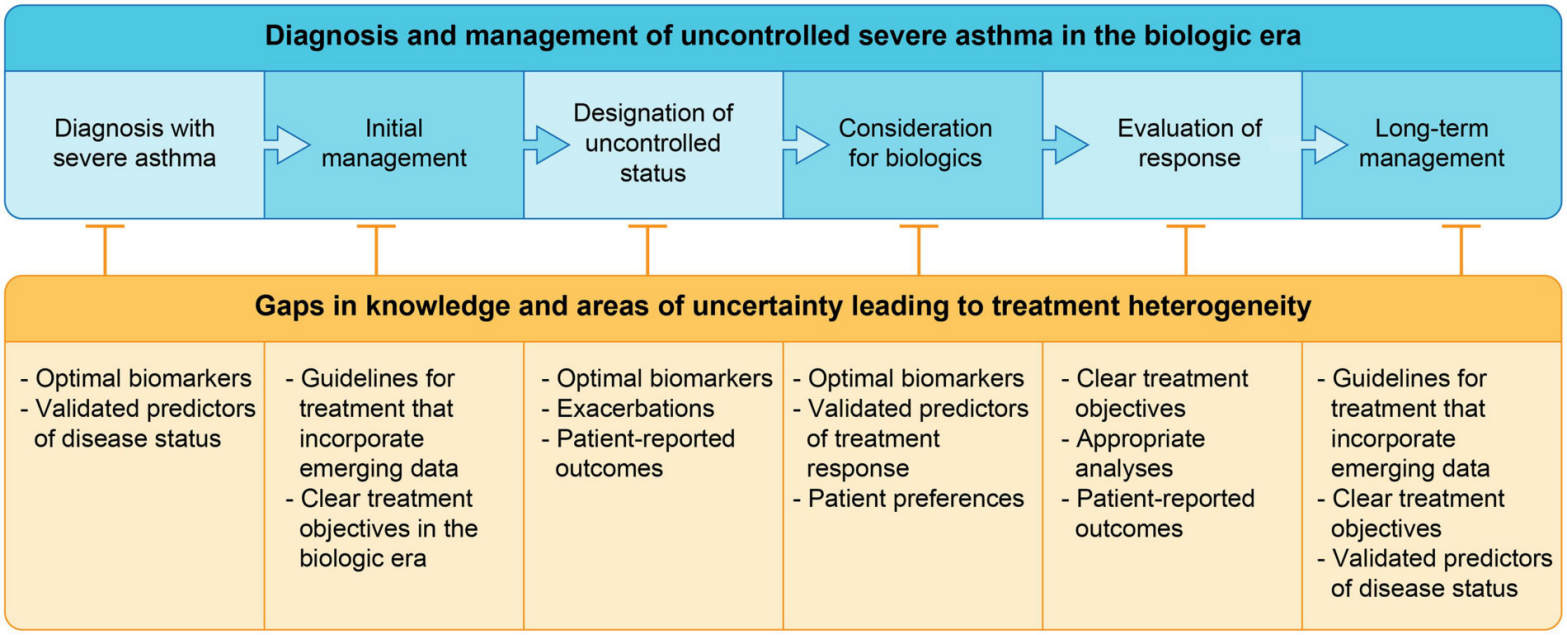
Diagnosis and management of uncontrolled severe asthma in the biologic era. The blue flow chart provides an overview of how severe asthma is diagnosed, established as uncontrolled, and managed over the long term. The orange boxes below highlight areas of uncertainty or limited knowledge that impede accurate diagnosis and optimal treatment.

This section will review how uncontrolled severe asthma is currently treated, with a focus on biomarkers, and look at challenges in the treatment landscape (Figure 1).

### 2.1 Current management approaches

For type 2-high severe asthma—defined in the GINA guidelines as asthma with one or more of the following criteria: blood eosinophil counts, ≥150 /μL; FeNO, ≥20 ppb; sputum eosinophils, ≥2%; and clinically allergen-driven disease—non-biologic approaches are recommended first, including further assessment of treatment adherence, increasing inhaled corticosteroids for 3–6 months, and prescription of a non-biologic treatment (3). When a biologic therapy is available and affordable, various tests and criteria are used to determine the most suitable option. Anti-IgE (omalizumab) is considered based on evidence of sensitization to inhaled allergens, total serum IgE levels and body weight, and the number of exacerbations within the last year. Indications for anti-IL-5 (mepolizumab or reslizumab) or anti-IL-5R*α* (benralizumab) include the number of severe exacerbations in the last year and blood eosinophil counts. Anti-IL-4R*α* (dupilumab) is considered based on evaluation of the number of exacerbations in the last year and either type 2 biomarker levels (blood eosinophil counts or FeNO) or the requirement for maintenance of oral corticosteroids. The number of severe exacerbations in the last year is typically used to indicate anti-thymic stromal lymphopoietin (TSLP; tezepelumab). Additional considerations for selecting a biologic include availability, dosing intervals, delivery routes, costs, and patient preference expressed as part of a shared decision-making process (1). A period of 4–6 months is typically recommended to evaluate patient response to the chosen biologic.

If there is no evidence of type 2 inflammation, anti-IL-4Rα or anti-TSLP may still be indicated, but non-biologic add-on treatments are also indicated. As stated in the GINA guidelines (3), biologics should be considered only after confirming that inhalation therapy is being appropriately administered with current controllers. The choice of biologics should also be made with consideration for the presence or absence of comorbidities for which they are indicated, such as chronic sinusitis. Although a flowchart is used in the guidelines (3), selecting a biologic is often not straightforward in actual clinical practice. This is especially true for cases with type 2 inflammation, in which overlapping features are frequently present. Biomarkers are among the currently available tools for guiding the choice of biologic therapy for severe uncontrolled asthma.

### 2.2 Biomarkers: established and emerging

Clinical studies have established biomarkers that can facilitate disease phenotyping and predict response to treatment as well as disease severity and exacerbations (9, 23). There are varying levels of evidence for these markers, and their utility depends on appropriate clinical application in the context of diagnosis, airway inflammation, disease control, and other areas related to treatment (24). Elevated blood eosinophil counts, FeNO, and serum IgE levels are commonly used in the clinic to determine a suitable course of treatment with biologics (3). Additional biomarkers of type 2 asthma include IL-4, IL-5, IL-13, IL-25, IL-33, thymus and activation-regulated chemokine, periostin, TSLP, C-C motif chemokine ligand (CCL) 13, CCL17, granulocyte-macrophage colony-stimulating factor, pulmonary and activation-regulated chemokine, and eotaxin levels; sputum eosinophil counts; and tissue eosinophils (9, 25). However, not all of these biomarkers are widely used in the clinical setting. Of these biomarkers, eosinophil counts, FeNO, IgE, and periostin are considered predictors of response to biologics (26). For type 2 inflammation, as described in the GINA guidelines (3), blood eosinophil counts, IgE levels, and FeNO are reasonable markers to use in the clinic. Although periostin and sputum eosinophil levels are useful, they are not easy to evaluate in routine clinical practice. Additional markers beyond blood neutrophil counts should also be developed to assess neutrophilic inflammation.

#### 2.2.1 Established biomarkers

Studies have revealed a confounding issue in the use of biomarkers to guide treatment: biomarker levels change over the course of treatment with biologics (9). Indeed, biologics can mask existing biomarkers, including blood eosinophil count, FeNO, and serum IgE levels, making it more difficult to assess appropriate next steps in treatment. Lindsley et al. (9) recently thoroughly reviewed how biomarkers change from baseline levels over the course of treatment of allergic asthma with omalizumab, eosinophilic asthma with mepolizumab and benralizumab, and treatment of all asthma phenotypes with dupilumab and tezepelumab. Blood eosinophil counts largely dropped with treatment, except with dupilumab. A reduction in FeNO was observed for most biologics and particularly with anti-IL-4R*α*. Serum IgE levels dropped with omalizumab, dupilumab, and tezepelumab, but this was not reported for the other biologics. Both sputum and tissue eosinophils dropped with biologic treatment, but data for the other biomarkers were sparse, with many not reported.

Although anti-IL-5 and anti-IL-5Rα agents did not reduce FeNO in all patients with severe asthma, recent evidence suggested that anti-IL-5 agents can reduce FeNO levels, and that responders to the agents were more likely to achieve clinical remission after 12 months (27). A further study demonstrated that FeNO levels could be a biomarker of response to anti-IL-5 and anti-IL-5Rα agents, in terms of lung function and oral corticosteroid sparing (28). Additionally, the anti-IL-5Rα agent benralizumab improved blood eosinophil counts and FeNO levels (29). These findings support previous evidence regarding blood eosinophil counts and suggest that benralizumab may have an effect on FeNO levels that had not previously been observed. Further research is needed to confirm the effects of biologics on biomarkers and validate the use of biomarkers in the clinic, especially after or during biologic treatment.

In patients treated with biologics, type 2 inflammation may be suppressed, making it difficult to assess accurately over the treatment course. Therefore, baseline eosinophil counts and FeNO levels measured prior to the initiation of biologic therapy should also be taken into account. Another important consideration is that—in the era of biologics—high peripheral blood neutrophil counts have been associated with poor disease control (22).

#### 2.2.2 Emerging biomarkers

Numerous studies have suggested that micro RNAs (miRNAs) play a role in regulating asthma pathogenesis and can be used as biomarkers in severe asthma (Table 1). Indeed, several miRNAs have been used to identify type 2-related asthma (miR-1248, miR-26a, let-7a, and let-7d in the serum) and to distinguish type 2 and non-type 2 asthma phenotypes (miR-21-5p, miR-126-3p, miR-146a-5p, and miR-215-5p in serum exosomes) (30). In adults with severe eosinophilic asthma, 8 weeks after initiation of mepolizumab or reslizumab, miRNA-338-3p was elevated, suggesting it could serve as a biomarker of the early response to anti-IL-5 agents (31). Additionally, benralizumab treatment altered levels of miR-1246, miR-5100, and miR-338-3p in adults with severe eosinophilic asthma, as well as levels of miR-21-5p. A recent study showed that miR-7-5p had the highest predictive accuracy for benralizumab effectiveness (32). Soccio et al. found that intracellular miR-21 levels are elevated in the serum of patients with severe asthma, and that miR-21, miR-223, and let-7a levels were higher in the exosomes of patients with severe asthma than in controls (33). A growing body of evidence has emerged for the role of miRNAs in asthma, suggesting that they may be useful in the diagnosis and phenotyping of asthma and the selection of biologics. More research into and validation of miRNA approaches are required before they can be used in clinical practice. These will be necessary to establish optimal protocols and reduce inter-laboratory variation. miRNA-based methods are likely to advance personalized medicine, but as we move toward greater standardization in the field, we must also consider access to these approaches and associated costs.

**Table 1:** miRNAs and other emerging biomarkers for severe asthma and treatment responsiveness.

Emerging biomarkers	Sample source	Marker for	Biologic	References
miR1248, miR-26a, let-7a, let-7d	Serum	Type 2-related asthma	–	(30)
miR-21-5p, miR-126-3p, miR-146a-5p, miR-215-5p	Serum exosomes	Differentiating type 2 versus non-type 2 asthma	–	(30)
miRNA-338-3p	Serum	Early response to anti-IL-5 agents	Mepolizumab, reslizumab	(31)
miR-7-5p	Serum	Benralizumab effectiveness	Benralizumab	(32)
miR-21, miR-223, let-7a	Serum exosomes	Severe asthma	–	(33)
Thioredoxin	Blood transcriptomic dataset	Severe asthma, immune infiltration	–	(34)
Coagulation factor V	Blood transcriptomic dataset	Severe asthma, immune infiltration	–	(34)

Recent studies have developed additional approaches to the diagnosis and monitoring of asthma and the biologic treatment response. The novel diagnostic gene markers thioredoxin and coagulation factor V, and associated miRNAs, were able to identify severe asthma (34) (Table 1). Skin sebum sampling for RNA analysis was identified as a method to monitor response to treatment with benralizumab in patients with severe asthma (35). Additionally, a volatile biomarker signature of sputum eosinophilia has recently been identified (36).

These studies present novel tools that are being developed to make severe asthma diagnosis and management more precise. However, additional validation in large-scale studies will be needed to bring these tools into common practice. As evidence accumulates, newly validated biomarkers should be incorporated into local and international guidelines, allowing them to be widely adopted in clinical settings. While biomarkers in the sputum and sebum are also important, blood-based biomarkers are likely to be preferred in clinical practice owing to their suitability for widespread testing.

### 2.3 Treatment heterogeneity in severe asthma

Although the expansion of treatment options in the biologic era has benefited patients, it has also introduced new challenges in disease management. A Delphi consensus study on management approaches, treatment decisions, biomarkers, and timing of biologic use in developing countries identified good consensus on some issues, including the use of some of the most common biomarkers for the diagnosis of severe asthma and treatment with biologics (37). However, many of the issues queried did not achieve a consensus view, highlighting the need for further research and the incorporation of more detailed criteria in guidelines. The study did not explore specifics, such as cut-off values, which could reveal the extent of treatment heterogeneity in these countries. Broader large-scale studies are needed to probe how biomarkers are utilized, to guide treatment decisions across the globe. Only when the underlying discrepancies are identified can they be addressed through further study and clearer guidance.

Furthermore, despite the existence of guidelines, inconsistencies in the evaluation and use of commonly accepted biomarkers and indicators for the prescription of biologics remain. A recent study found that when presented with seven real-life cases of asthma managed with a biologic (omalizumab, mepolizumab, reslizumab, benralizumab, or dupilumab), 16 physicians in Quebec, Canada, showed weak agreement on the timing and type of treatment selected (38). These findings suggest that notable treatment heterogeneity exists even within the same geographical region, necessitating clearer guidance for optimal treatment. Understanding the forces driving disparate treatment decisions within geographical regions may provide insight into the relative contributions of factors including access, cost, and awareness of treatment options. Moreover, further research in this area may identify as-yet unappreciated factors that influence physician choice.

Because current biologics share similar therapeutic targets, the chosen treatment strategy often depends on the attending physician. Therefore, in addition to identifying new biomarkers, it is essential to develop therapeutic agents with new mechanisms of action. Given the difficulties in evaluating efficacy over a short period of time, it is necessary to define the appropriate timing for assessing the effectiveness of biologic therapies. Moreover, biomarkers are needed to evaluate treatment efficacy in combination with questionnaire-based assessments.

## 3 Emerging tools to guide asthma treatment in the biologic era

**Figure 2:**
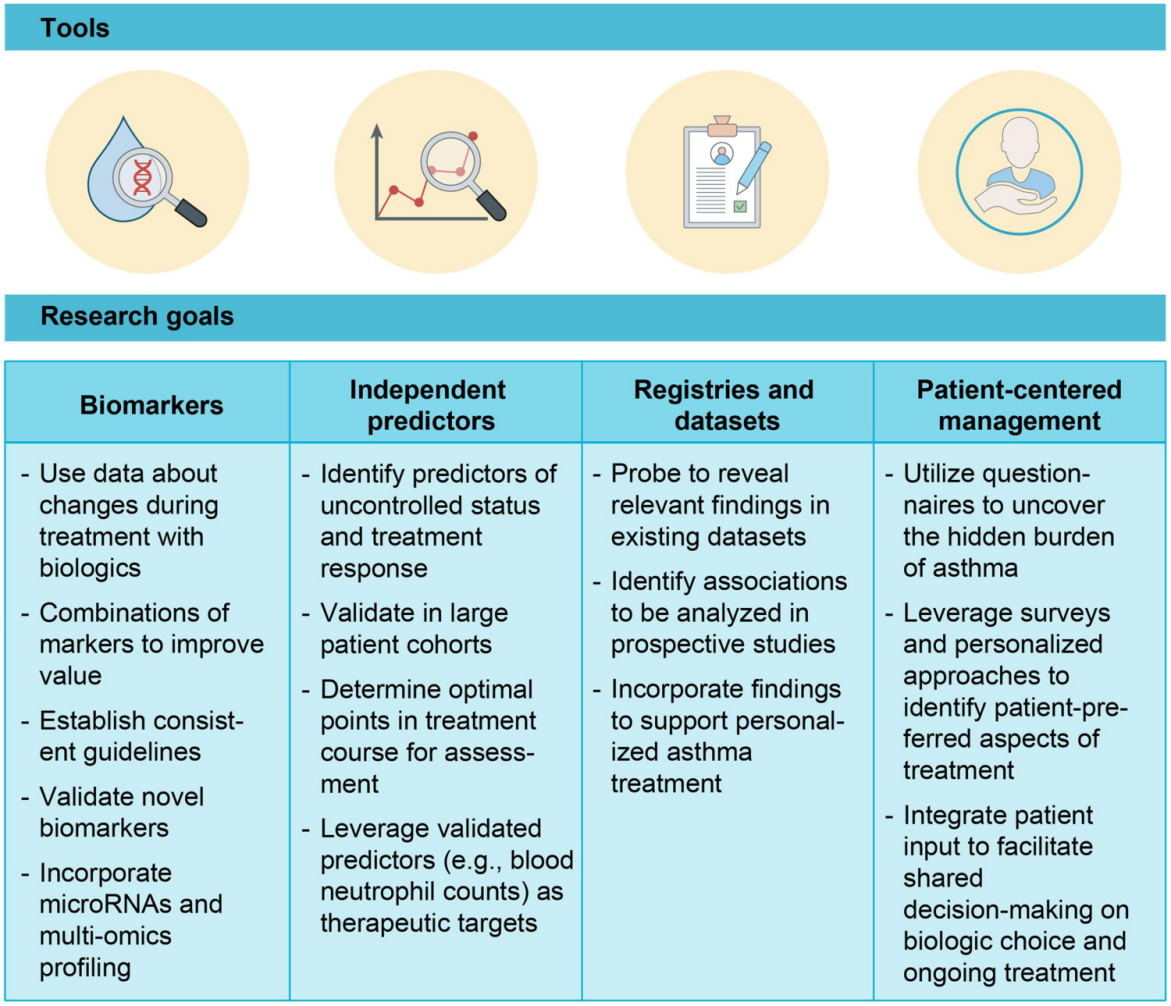
Goals for using available tools to guide decision-making and minimize treatment heterogeneity in uncontrolled severe asthma. While numerous options are available to treat severe asthma in the age of biologics, no clear, universal approach to management has been established. We can better leverage biomarkers, independent predictors, registries and datasets, and patient input to inform treatment decisions, monitor effectiveness, and improve patient outcomes.

This section will provide an up-to-date look at predictors of disease status and treatment response, and consider emerging techniques to assess treatment response for severe asthma (Figure 2).

### 3.1 Independent predictors of uncontrolled status and response to biologics

Balasubramanyam et al. (7) found that asthma biologics show great effectiveness in real-world studies. Their findings and analytical methods could guide future efforts in precision medicine with personalized treatment plans.

In a recent study using multivariate analysis, we identified blood neutrophil count as an independent predictor of uncontrolled asthma status, regardless of the use of biologics (22) (Table 2). Importantly, these findings suggest that blood neutrophil count may remain a useful metric throughout the diagnosis and treatment process. As noted above, approximately 40% of cases of severe asthma in Japan are classified as uncontrolled, despite advances in treatment (5), emphasizing the potential value of validated and clinically applicable independent predictors of status that could provide insight into the management of such uncontrolled cases.

**Table 2:** Independent predictors of severe asthma status or response to biologics.

Independent predictor	Biologic	Predictor of status or response	Evidence	Reference
Blood neutrophil count	Regardless of use	Uncontrolled status	Clinical	(22)
Blood eosinophil count	Benralizumab	Biologic response	Clinical	(39)
FeNO	Benralizumab	Biologic response	Clinical	(39)
Airway obstruction	Benralizumab, omalizumab, mepolizumab	Biologic response	Clinical	(15)
Mucus plug score	Benralizumab, omalizumab, mepolizumab, dupilumab, reslizumab, tezepelumab	Biologic response	Clinical	(40)
FeNO 50	Benralizumab, dupilumab	Clinical remission	Clinical	(41)
Serum and urine neurotoxin	Mepolizumab, reslizumab, or dupilumab	Biologic response	Clinical	(42)

FeNO, fractional exhaled nitric oxide; FeNO 50, FeNO at a flow rate of 50 mL/s.

Another study provided evidence that the baseline values of the established biomarkers blood eosinophil count and FeNO can be used as independent predictors of response to benralizumab (39). Further recent studies have indicated that additional factors may have utility as predictors of response to biologics. In a cohort of patients with severe asthma treated with omalizumab, benralizumab, and mepolizumab, airflow obstruction was a predictor of non-complete response to biologics (15). Additionally, a higher mucus plugging score in biologic-naïve patients predicted a greater clinical and pulmonary function response to biologic therapy after 4 months (40). After 1 month of treatment with biologics, a reduction in FeNO at 50 mL/s was associated with a higher rate of clinical remission after 12 months (41). In patients with severe asthma who received mepolizumab, reslizumab, or dupilumab, serum and urine eosinophil-derived neurotoxin levels were comparable to blood eosinophil counts in predicting treatment response (42). While these studies provide additional data on independent predictors of treatment response, gaps remain in the literature. More comprehensive studies covering a wide range of predictors and all available biologics are needed to better inform severe asthma treatment.

Data from 21 countries suggest that the accuracy of predictors of response varies owing to heterogeneous patient responses to biologics and is dependent on the type of assessment (43). There was a positive association of higher blood eosinophil counts and type 2-related comorbidities with response criteria for long-term oral corticosteroids, disease control, and lung function. Notably, many patients who respond to biologic therapy still have residual symptoms, so different methods to assess response will yield different results, highlighting the need for more precise methods of monitoring treatment response.

### 3.2 Molecular tools to monitor and guide treatment

Among emerging tools, molecular techniques may have substantial power to guide severe asthma management. Single-cell RNA sequencing of blood cells from patients with severe eosinophilic asthma who received mepolizumab, reslizumab, or dupilumab uncovered changes in the composition of classical monocytes, some T cell subsets, and individual signaling pathways (44). A multi-omics monitoring approach for inflammatory metabolites and proteins in patients with severe asthma who received mepolizumab or omalizumab, paired with multivariate analysis, revealed several potential biomarkers associated with clinical improvement on mepolizumab (45). It will be important to evaluate these biomarkers alongside existing, established markers, as they provide objective indicators of inflammation that cannot be assessed through questionnaires. However, we believe that it would be difficult to determine disease control status based on a single novel biomarker. A combination approach that incorporates biomarker evaluation and questionnaire-based assessment is likely to offer the greatest insight into treatment efficacy.

### 3.3 The use of questionnaires and surveys to incorporate patient preferences and patient-reported outcomes in clinical decision-making

To complement the evaluation of biomarkers and independent predictors, clinicians will need to incorporate additional tools to guide the treatment of uncontrolled severe asthma. Beyond laboratory testing and biomarker assessment, measuring patient-reported factors may be an important element of clinical care. Recent findings suggest that patient-reported outcomes are associated with the number of exacerbations and have only a weak association with clinical characteristics (46), emphasizing the need to incorporate patient input into assessments of treatment to achieve a holistic understanding of patient outcomes. A recent Delphi questionnaire study that included patients and clinicians identified a set of patient-reported outcome measures that could aid clinical practice, but also identified several barriers to their use, related to the health system, care providers, and patients (47). Some measures were deemed infeasible for clinical use, while others awaited real-world clinical validation.

A recent review echoed these concerns and highlighted the need for the validation of these tools in the clinic and better utilization to improve patient outcomes (48). A discrete choice experiment on patient vs. clinician preferences for biologic treatments for uncontrolled severe asthma revealed differences between the two groups, with patients placing greater emphasis on personal experience and location of administration (49). Clinicians expressed greater concern for treatment compliance and cost. In my own experience, the cost of the drug and the option for self-injection appear to influence a patient’s decision-making process.

Shared decision-making is a process that incorporates or acknowledges patient preferences in care decisions (50). It relies on clear communication of objective information, both risks and benefits, and involves the patient and, where applicable, their family. A recent study suggested that shared decision-making did not correlate with inhaled medication adherence in patients with asthma (51). Although the use of biologics raises concerns about reduced adherence to inhaled medications, further research is needed to clarify the relationship between biologic use and treatment adherence in severe asthma.

I have found that some patients with severe asthma are strongly opposed to injectable and high-cost medications. Moreover, some physicians are willing to prescribe biologics, while others are more hesitant. In regions with a limited number of respiratory specialists, there is concern that the introduction of biologics may be delayed. Although few patients with severe asthma—unlike those with cancer—currently seek second opinions in Japan, it will be important to provide such opportunities in the future. Regional differences in the incidence of asthma exacerbations in Japan are also likely to influence available resources and patient attitudes toward management (52).

## 4 Future directions to improve treatment

On the basis of my clinical experience, I have identified several elements of asthma management that can be improved, standardized, or complemented to enhance disease outcomes and patient quality of life. Some of my suggestions can be implemented immediately, whereas others require additional research and the development of further tools.

### 4.1 Standardization of care with current tools

Changes to clinical practice using current knowledge can be leveraged to improve the treatment of uncontrolled severe asthma. At this stage, it is appropriate to assess asthma control using the number of exacerbations in the past year, questionnaire scores, and type 2 biomarkers (i.e., blood eosinophil counts and FeNO), as recommended in the GINA guidelines (3). This standardization of care relies on existing methods and would contribute to patient care and ongoing research efforts.

Furthermore, existing and emerging databases and registries can be used to analyze retrospective data to guide treatment decisions and future research efforts (5, 53). Indeed, recent studies have demonstrated that data mined from the German Asthma Net registry can guide clinical decisions for patients with a history of smoking (54, 55).

**Figure 3:**
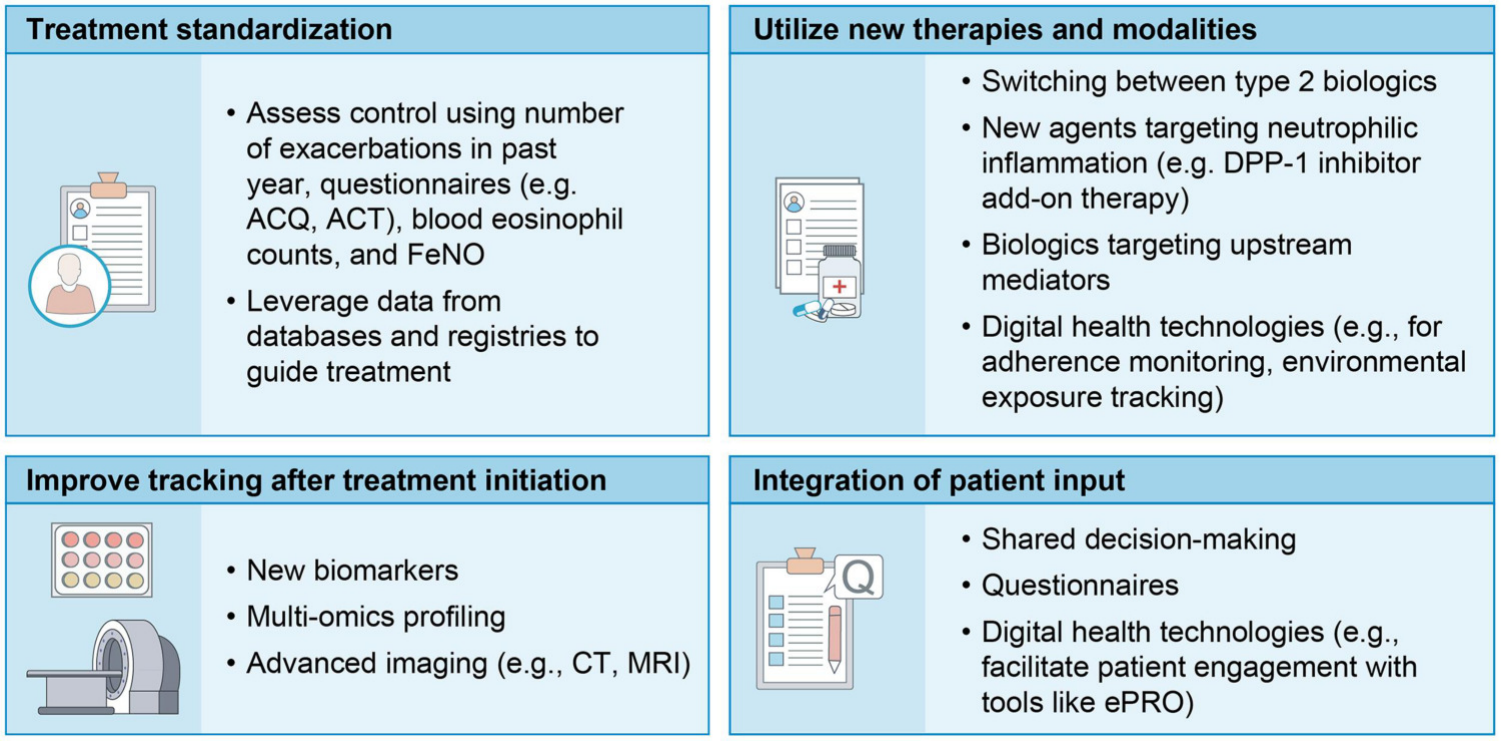
Looking to the future of uncontrolled severe asthma management. To further improve clinical decision-making and build on recent successes in severe asthma treatment, we should target four key areas of management. Treatment should be standardized based on clinical evidence to reduce heterogeneity, new therapeutic approaches should be incorporated, monitoring of asthma after treatment initiation should be improved using new biomarkers and imaging modalities, and patient input should be at the center during a shared decision-making process. ACQ, Asthma Control Questionnaire; ACT, Asthma Control Test; CT, computed tomography; DPP-1, dipeptidyl peptidase 1; FeNO, fractional exhaled nitric oxide; MRI, magnetic resonance imaging.

Looking to the future, several areas of further research are needed to address gaps in the literature and move toward optimal management of severe asthma with biologics (Figure 3).

### 4.2 Implementation of emerging modalities

Alongside the development of new biologic agents, there is a need to develop biomarkers that can guide the selection of appropriate biologics. In clinical practice, biologic therapies targeting type 2 inflammation have significantly improved outcomes in patients with severe asthma. However, an important challenge arises when biomarkers traditionally used to guide treatment, including blood eosinophils, FeNO, and serum IgE, are suppressed by therapy. Asthma may remain poorly controlled despite apparent attenuation of type 2 signals. In such cases, clinicians face uncertainty in determining the next step in care. At present, treatment decisions in this scenario rely primarily on clinical outcomes, such as exacerbation frequency, oral corticosteroid dependence, and patient-reported symptom control, rather than on biomarker guidance.

Switching between biologics with different targets—for example, from anti-IL-5 to anti-IL-4Rα agents—is often considered. This is particularly true when comorbidities such as chronic rhinosinusitis with nasal polyps or atopic dermatitis suggest an alternative inflammatory pathway. If asthma control remains difficult even with robust suppression of type 2 inflammation, agents targeting neutrophilic inflammation may be useful in the future. For example, dipeptidyl peptidase 1 (DPP-1) inhibitors are likely to be approved for bronchiectasis in the future. They may also be candidates for the treatment of asthma complicated with bronchiectasis by neutrophilic inflammation (56). Furthermore, plasminogen activator inhibitor-1 (PAI-1) levels were elevated in patients with asthma and increased with disease severity (57). In preclinical studies, PAI-1 inhibition suppressed neutrophilic airway inflammation in a chronic obstructive pulmonary disease model (58). It also ameliorated both airway inflammation and airway remodeling in a chronic asthma model (59). Therefore, PAI-1 inhibition represents a potential therapeutic strategy for neutrophilic inflammation in severe asthma.

For patients with evidence of non-type 2 inflammation, adjunctive approaches such as long-term macrolide therapy or treatment with long-acting muscarinic antagonists may be appropriate. Biologic agents targeting non-type 2 inflammation are not yet available. However, novel biologics targeting upstream mediators, such as TSLP or IL-33, may further expand treatment options for patients who remain uncontrolled despite suppression of type 2 inflammation. Emerging tools hold promise for addressing this gap.

Multi-omics profiling, including miRNA analysis, of blood or airway samples may help to identify residual inflammatory endotypes even under biologic therapy. Advanced imaging techniques can reveal structural remodeling, which may necessitate non-biologic interventions (60). Furthermore, digital health technologies, including adherence monitoring (61) and environmental exposure tracking (62), can help differentiate treatment resistance from modifiable behavioral or environmental factors.

### 4.3 Better integration of patient input

Incorporating patient preferences into treatment decisions, such as desired symptom relief, tolerance of potential adverse effects, injection frequency, or lifestyle considerations, can enhance adherence and overall quality of life. Shared decision-making provides a structured framework to integrate these preferences with evidence-based clinical data. For example, selection among biologics, such as anti-IL-5 or anti-IL-4R*α* antibodies, can be guided by assessing residual inflammation markers and comorbidities such as nasal polyps and atopic dermatitis, and coincide with patient priorities regarding convenience and side-effect profiles. Emerging tools, including digital health technologies and patient-reported outcome measures, may further refine individualized treatment strategies. Integration of clinical biomarkers with patient-centered data into decision support systems could allow for more precise, personalized therapy, particularly in cases where standard biomarkers are masked by ongoing biologic therapy. Overall, optimal management of severe asthma depends on the harmonization of objective clinical indicators with patient preferences, ensuring that treatment decisions are both evidence-based and aligned with individual values, thereby maximizing clinical benefit and adherence.

## 5 Conclusions: treatment and research frontiers

The advent of biologics has changed severe asthma treatment choices and goals toward a more targeted approach aimed at controlling the underlying causes of asthma and a focus on achieving remission, rather than short-term relief. Biomarkers are useful to inform treatment choices in the biologic era, but may not be used to their fullest extent. The GINA guidelines advise the use of only a handful of biomarkers, despite experimental evidence for the utility of additional markers. Further research to validate novel biomarkers and updates to guidelines and clinical practice will be needed to improve the treatment of uncontrolled severe asthma.

Beyond biomarkers, independent predictors of disease status and treatment response will be essential to optimizing the management of uncontrolled severe asthma. A rigorous statistical approach will be needed to identify and validate independent predictors to guide clinical decisions. To overcome existing treatment heterogeneity and improve patient outcomes, an integrated approach will be necessary, incorporating biomarkers, independent predictors, patient preferences, and patient-reported outcomes.
